# Involvement of persons with lived experience of a prenatal diagnosis of congenital heart defect: an explorative study to gain insights into perspectives on future research

**DOI:** 10.1186/s40900-016-0048-5

**Published:** 2016-12-15

**Authors:** Tommy Carlsson, Ulla Melander Marttala, Barbro Wadensten, Gunnar Bergman, Elisabet Mattsson

**Affiliations:** 1 Department of Public Health and Caring Sciences, Uppsala University, BMC Husargatan 3, Box 564, S-75122 Uppsala, Sweden; 2 Department of Scandinavian Languages, Uppsala University, Uppsala, Sweden; 3 Department of Women’s and Children’s Health, Karolinska Institutet, Stockholm, Sweden; 4 Department of Health Care Sciences, Ersta Sköndal University College, Stockholm, Sweden

**Keywords:** Congenital heart defects, Needs assessment, Patient participation, Prenatal diagnosis

## Abstract

**Plain English summary:**

Ultrasound examinations during pregnancy have led to an increased number of detected heart defects in fetuses. Pregnant women and their partners are often unprepared for these news, and experience several difficulties following the diagnosis. We asked persons with personal experience to participate in group discussions about relevant future research topics. The discussions revealed that future research should investigate supplemental written information or follow-up appointments with health professionals. Researchers were also encouraged to focus their efforts on structures that offer emotional support. The emotional support could be from those that share similar experiences, or additional support from a health professional. The results of this study illustrate the need for researchers to continue their work to test ways to support persons faced with these diagnoses.

**Abstract:**

**Background**

A prenatal diagnosis of a congenital heart defect in the fetus is a traumatic life event for pregnant women and their partners. Previous research indicates a need for research that takes steps to support these individuals following the diagnosis. Patient and public involvement is a proposed method of identifying relevant research topics, leading to patient-focused research protocols and relevant support interventions.The overarching aim of this study was to gain insights into relevant future research topics among persons faced with a pre﻿na﻿tal diagnosis of congenita﻿l heart defect in the fetus.

**Methods**

One group of parents to prenatally diagnosed children with a congenital heart defect (*n* = 5) and one group of individuals with experience of termination of a pregnancy following a prenatal diagnosis of a congenital heart defect (*n* = 5) were purposefully recruited. Each group of representatives was involved in a face-to-face focus group discussion, analyzed through qualitative content analysis.

**Results**

The representatives suggested a need for future research that addresses informational support in the forms as supplemental written information or follow-up consultations. Moreover, interventions that offer emotional support were suggested, in the forms of peer support or additional professional psychosocial support.

**Conclusion**

Several interventions were suggested by patient representatives, indicating a need for multiple intervention studies to be conducted in the context of a prenatal diagnosis of a congenital heart defect in the fetus. We recommend that future studies test supplemental written information, follow-up consultations, peer support, and additional professionals psychosocial support following the diagnosis.

## Background

The introduction of obstetric second-trimester ultrasound examinations of fetuses has improved the overall detection rate of major congenital heart defects (CHD) [1]. Pregnant women view the examination with optimistic expectations [2, 3] and feel unprepared when faced with a detection of a fetal anomaly [2–6]. A prenatal diagnosis of a fetal anomaly is for expectant parents a traumatic life event [6], involving acute grief reactions [7] and considerable psychological distress [7–9]. Depending on state laws, pregnant women may have the option to terminate the pregnancy, a decision that raises ethical dilemmas [10]. To reach an informed decision, sufficient information that covers many different topics is required [11, 12]. Recent explorative studies have illustrated the complexity of being faced with a prenatal diagnosis, to adequately offer support for these individuals. For example, expectant parents faced with a prenatal diagnosis of fetal anomaly describe overall satisfaction with the information provided by the specialist specifically about the defect [11, 13, 14], but want more information about various related topics such as available support groups, specialist treatment, raising a child with a disability, and recommendations for Internet sources [15, 16]. Moreover, they often experience difficulties sorting out, interpreting, and remembering the information provided [11], indicating a need for improvement in the delivery of information following the diagnosis. Additionally, previous research indicates a need for research that addresses psychological difficulties and need for support following the diagnosis [12, 17, 18].

Previous research has reported a mismatch between research and the interests of patients, suggesting that research agenda is not always in line with patient needs. Studies that involve patients in decisions about research have shown that patients suggest research about topics more closely related to their experienced issues, in comparison to the topics suggested by researchers or other stakeholders. Such examples of mismatched perspectives include sexual dysfunction caused by antipsychotic medication [19], education for patients with osteoarthritis [20] and adverse effects of inhaled steroids among asthma patients [21]. To confront this, patient and public involvement (PPI) has been introduced as a commitment to empower individuals and communities to have a greater impact over health care research [22]. In this study, we adhere to the definition of PPI as stated by the INVOLVE advisory group: “Research being carried out ‘with’ or ‘by’ members of the public rather than ‘to’, ‘about’ or ‘for’ them” [23]. PPI can have varying degrees of public involvement, including consultations, collaborations and user-controlled research [24]. Through consultations, members of the public are asked for their views to guide decision-making, for example regarding which research topics to focus on. Consultations present an appropriate starting point for projects, and enable researchers to gain insights through discussions when exploring sensitive and difficult subjects [23]. Thus, PPI has the potential to help define relevant questions and outcomes in the initial stages of research [22, 25, 26]. In addition, PPI presents an opportunity for researchers to gain insights into specific health needs, leading to more patient-focused research protocols [25]. PPI approaches within maternity care have revealed that women prioritize research topics concerning antenatal care as well as communication and information [27], raising questions about which topics are prioritized by those with experience of a prenatal diagnosis. Consequently, more research is needed to gain a deeper understanding of appropriate ways to guide future research within this area. With this study, we set out to generate novel ideas about research topics prioritized when a congenital anomaly is detected in the fetus. Thus, the overarching aim of this study was to gain insights into relevant future research topics among persons faced with a prenatal diagnosis of congenital heart defect in the fetus.

## Methods

### Study design

In accordance with the multidimensional framework for public involvement in health services presented by Oliver et al. [24], this study positions itself as research that invites individual lay people to participate in face-to-face focus group consultations about research. In accordance with the statement from the INVOLVE advisory group [23], we decided to conduct group consultations because of their appropriateness as a starting point for research projects and the overall lack of knowledge about research topics prioritized by persons with experience of a prenatal diagnosis of congenital heart defect in the fetus. Overall, our positions and reasons behind the consultations were inductive, with the general purpose of gaining insights grounded in the perspectives and preferences of the representatives we invited. Consequently, two PPI groups were formed: one group with parents to prenatally diagnosed children with CHD and one with persons who terminated a pregnancy following a prenatal diagnosis of CHD. The participants in the groups are hereafter referred to as patient representatives.

### Study context

In Sweden, all pregnant women are offered a second-trimester obstetric ultrasound examination. The primary medical purposes of the examination are to assess the gestational age, detect twin pregnancies, investigate the anatomy of the fetus and to determine the location of placenta. Most women accept the offer to screen their pregnancies [28]. Women with a fetus suspected of having a CHD are referred to a fetal cardiologist for ultrasound examination and consultation. Based on the findings and precision of the examination, information on a broad variety of topics is offered orally and through drawings. The risks of associated anomalies are highlighted, and if needed additional fetal investigations are offered.

According to Swedish legislation women have the right to terminate the pregnancy up until 18 weeks and 0 days of gestation. When the pregnancy is beyond 18 weeks and 0 days of gestation, termination needs to be approved by the National Board of Health and Welfare. In clinical praxis, approval is seldom given after 22 weeks of gestation. If the woman chooses to terminate the pregnancy, a social worker assists her with the application to the National Board of Health and Welfare and offers psychosocial support. In Sweden, pregnancy terminations after 12 weeks of gestation typically involve induced labor and vaginal delivery of the fetus. After pregnancy termination, follow-up is offered at the fetal medicine unit and, if needed, at the fetal cardiology unit as well.

When the pregnancy is continued, fetal cardiology follow-up is offered every 4-6 weeks in addition to the fetal medicine program. The purpose of these visits is to monitor the progression of the CHD, prepare the couple for the delivery, and optimize the planning of the perinatal management.

### Patient representatives

#### Recruitment

**Fig. 1:**
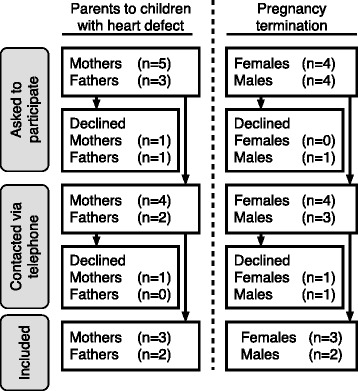
Recruitment process

Patient representatives were purposefully recruited [29], to strive for maximum variation with regard to country of birth, educational level and age. The patient representatives were recruited via the units for fetal and pediatric cardiology at Uppsala University Hospital, Uppsala and Astrid Lindgren Children’s Hospital, Stockholm, Sweden (Fig. 1). The study was approved by the Regional Ethical Board in Uppsala, Sweden (Approval number 2014/504/1). Oral and written information was given to all potential patient representatives and written consent to participate in the study was collected before enrollment. They were given information that their participation was completely voluntary, and that they were free to end their participation without having to state any reason for this. The groups worked separately and all patient representatives received SEK 1,500 after taxes, as compensation for travel and lost earnings.

#### The parent group

The last author was responsible for recruitment of parents through her clinical network. Eight parents to four prenatally diagnosed children were asked to participate in the PPI group. One couple declined participation because they feared that painful memories from the child’s hospitalization would be rekindled by group sessions. Six parents agreed to be contacted by the first author via telephone for further information. However, when contacted, one mother refrained from participation as she felt it was enough with one parent from the family. Thus, three mothers and two fathers consented to participate in the PPI group.

#### The termination of pregnancy group

The first author was responsible for recruitment of individuals with experience of termination of pregnancy following a prenatal diagnosis of CHD. Potential patient representatives were identified when they took part in a previous interview study [11]. Eight (four females and four males) out of 15 participants from the interview study were asked to participate. One male declined to be contacted for further information because of lack of interest. Four females and three males agreed to be contacted for further information. When contacted, one female and one male declined because of lack of time. Thus, three females and two males consented to participate in the PPI group.

### Characteristics of included patient representatives

Table 1 presents characteristics of the included patient representatives. All pregnancy terminations had been medically induced labor and vaginal deliveries. Among the parents, the age of their child with heart defect ranged between two and seven years. The fetal diagnoses included atrioventricular septal defect with associated trisomy 21 (*n* = 2 who terminated the pregnancy), coarctation of the aorta (*n* = 1 parent), critical aortic stenosis (*n* = 2 who terminated the pregnancy), Ebstein’s anomaly and tricuspid dysplasia with multiple extracardial malformations (*n* = 1 who terminated the pregnancy), transposition of the great arteries and ventricular septal defect and pulmonary stenosis (*n* = 2 parents), and ventricular septal defect (*n* = 2 parents). The patient representatives had no previous contact with each other before the PPI groups started, except for two persons in each group who were couples.

**Table 1:** Characteristics of patient representatives who were parents to prenatally diagnosed children (*n* = 5) and with experience of termination of pregnancy (*n* = 5)

Characteristic	Category	Parents, *n*	Pregnancy termination, *n*
Age (years)	20-29	-	3
	30-39	2	1
	>39	3	1
Country of birth	Sweden	2	5
	Other	3	-
Born children	0	-	3
	1	2	2
	2	1	-
	>3	2	-
Highest education	Senior high school	1	1
	University/ College	4	4

### Data collection

In April and June 2015, one focus group discussion was held with each group. The discussions were guided by three main questions: “What difficulties did you experience following the diagnosis”, “What support needs did you experience following the diagnosis”, and “Do you have any suggestions on a relevant support intervention to test in future research”. The first and second authors attended the meetings. The first author moderated the discussion and wrote down notes on a whiteboard during the discussions. The second author asked questions and wrote handwritten notes. The discussions were digitally audio-recorded and transcribed verbatim. The discussions lasted 1 h 12 min for the parent group and 1 h 22 min for the termination group.

### Data analysis

The transcripts were subjected to qualitative content analysis [30], and analyzed utilizing Nvivo for Mac (QSR International Pty Ltd. Version 10.2.2). Initially, the transcripts were read repeatedly to gain an overall perspective of the content. Meaning units, defined as words, sentences or paragraphs containing aspects related to each other in content and context, were identified, condensed, and assigned a descriptive code. The codes were structured into categories, defined as collections of different codes that have a common subject. The first author was responsible for the primary analysis. The findings were discussed with the second and last author until consensus was reached.

## Results

The discussions revealed two categories of suggested interventions. Firstly, interventions that offer informational support were suggested, with the purpose to support difficulties with information uptake and retention. Secondly, peer support interventions were suggested, with the purpose to support psychological difficulties.

### Informational support

The representatives pointed out the considerable difficulties experienced with uptake and retention of information offered during consultations with health professionals. To meet the needs related to these difficulties and promote comprehension, they suggested interventions that provide informational support. According to the representatives, these interventions could either be supplemental written information, or follow-up consultations with health professionals. Moreover, representatives called attention to informational support interventions specifically developed to support those who terminate the pregnancy by offering information about second-trimester pregnancy terminations. Table 2 presents suggested interventions for informational support with illustrative quotes from the representatives.

**Table 2:** Suggested interventions for informational support with illustrative quotes

Suggested interventions	Illustrative quotes
Supplemental written information	1. I think it feels good to be able to get a note or brochure [containing information]. (Father 1)
	2. I thought that everything was completely clear when I sat there and got the information, but then I came home and was going to tell my grandmother, maybe, or someone else about it, and then I couldn’t remember and I didn’t know how it was. (Mother 2)
	3. Somethat that would really be a help in any case would be to get a piece of paper. Perhaps just one or two pages of general information about the heart but also where you can look for information. (Mother 3)
	4. All the information about what to apply for and what help there is and things like that comes too late. (Father 2)
	5. I think it feels good to be able to have a leaflet like that, a brochure. […] Maybe you sit at home and read it, you maybe go to your grandparents and take it with you. You don’t have to go on the Internet. (Mother 3)
	6. For me an information website on the Internet would probably have been enough […] Purely information, that is. (Female 2, pregnancy termination)
	7. We were very happy anyway that we could go home and read the information on the Internet. (Male 2, pregnancy termination)
	8. I don’t remember half of what he [the doctor] said, mind you. (Female 1, pregnancy termination)
	9. Yes, you can find all kinds of strange things that you shouldn’t read [on the Internet]… (Female 3, pregnancy termination)
	10. Information on a website is a good start. (Male 2, pregnancy termination)
Follow-up consultation	1. Often when you read, questions arise and sometimes you don’t have any more contact with the doctors before you have to reach a decision. (Mother 2)
	2. Then perhaps you could meet a few days later and go through it again, because you digest it and take things in and interpret. And you distort things a bit. (Mother 3)
	3. I would have appreciated the same information repeatedly […] A second consultation with a physician or a specialist. […] A whole lot of questions usually arise, the more you read about it. (Father 1)
	4. Because I noticed when I was there afterwards that I would have preferred a follow-up talk with a midwife or something like that. (Female 2, pregnancy termination)
	5. We met two doctors and I think that was why we could take it in better […] The second time we were more open because we had had time to think a bit. (Female 1, pregnancy termination)

#### Supplemental written information

The representatives suggested a need for interventions that offer supplemental written information, either in the form of a website or as a brochure. A website that includes supplemental information was considered to be highly accessible and needed. They described that it could provide an opportunity to repeat information at home following the diagnosis. Representatives wanted the information website to include trustworthy information, written by specialists. The parents also suggested a supplemental information brochure as an alternative to an information website. Their opinions were that a brochure would be easier to have at hand and be something concrete, in comparison to a website.

#### Follow-up consultation

Representatives suggested follow-up counseling sessions with specialist health professionals, to offer repeated information and present an opportunity to ask questions. They pointed out the importance of a follow-up counseling session when the initial shock has eased. A contact person within the health care system and a telephone hotline to reach a health professional was also suggested.

### Emotional support

Representatives described several psychological difficulties following the diagnosis. To deal with these, they pointed out the potential benefits of interventions that offer emotional support. According to the representatives, the emotional support could either be peer support from individuals with similar experiences, or additional professional psychosocial support. Table 3 presents suggested interventions for emotional support with illustrative quotes from the representatives.

**Table 3:** Suggested interventions for informational support with illustrative quotes

Suggested interventions	Illustrative quotes
Peer support	1. You can just offer that it exists. Just to know it exists. Then you yourself can decide if you want [to meet others with experience]. (Mother 1)
	2. The Swedish Childrens Heart Association [patient organization], they should also help with this. And unfortunately you get, you usually get all the information and everything after the child has been born. (Father 2)
	3. I think that contact where you can ask questions is good. And it doesn’t have to be a meeting in person. But there is also this Facebook page where everyone can ask questions, there are a lot of people who reply. You have a lot of thoughts and questions. (Mother 3)
	4. I would think before the first two weeks would be good maybe [to meet peers], because then you have… Mentally prepared, I think. (Father 1)
	5. Afterwards [after the termination] I would like to talk to someone who has gone through a similar experience. That would be very beneficial. (Female 1, pregnancy termination)
	6. I wanted to hear others who had gone through this, how things had gone for them afterwards, there were a lot of question marks. (Female 2, pregnancy termination)
	7. It was a wish I had. I would have liked, perhaps not exactly in the heat of the moment when things were at their worst, but after a while [to have talked with peers]. (Female 3, pregnancy termination)
Additional professional psychosocial support	1. And perhaps if you can get help afterwards, like counselors who help you with everything, both your soul and paper and social insurance and whatever. (Mother 2)
	2. Perhaps a telephone number you can ring 24 h a day, that is irrespective of what questions you have on your mind. (Father 2)
	3. When you go through these kinds of things, the least you should be offered is to talk with a social worker. And by that I mean to really talk, not just to sit down for fifteen minutes. (Female 1, pregnancy termination)
	4. I received really good help. I had a doctor who informed us that our fetus had this heart defect and who followed us the whole way and who even also phoned us afterwards and made an appointment for a talk, a little follow-up talk, how things are now, do you experience. So I experience that I have received that for my part. And it was worth a lot. I hope that everyone receives that. The support… (Female 3, pregnancy termination)

#### Peer support

Peer support was considered to be highly valued, and many representatives described a need for interventions that offer these kinds of supportive structures. The parents suggested an intervention to offer contact with other parents to children with CHD. Such structures would present opportunities to ask questions and to gain insights into the lives of families and children with CHD. Their opinions were that peer support should be offered a few days following the diagnosis and continued during the remainder of the pregnancy and also after the birth. The peer support could be either face-to-face, via telephone, or offered via the Internet.

Those who terminated the pregnancy suggested peer support from others with similar experiences of pregnancy termination, either in face-to-face settings or via virtual platforms. Such structures would present opportunities to talk about perinatal loss, grief and feelings after the termination. Consequently, the representatives described that these peer support structures should only be offered after the pregnancy termination has taken place. It was suggested that virtual peer support should be offered in secure and confidential platforms only available to individuals with similar experiences and decisions, to avoid encounters with critics.

#### Additional professional psychosocial support

The representatives suggested additional professional psychosocial support as an intervention for future research. To be offered counseling by a social worker or a nurse was considered to potentially be highly valuable. In addition to offering psychosocial support, the respondents also suggested that the social worker should be able to aid with administrative issues, such as health insurance for the expected child. Preferably, the social worker would be available for several repeated sessions, regardless if the pregnancy was continued or terminated. The respondents also mentioned that counseling sessions via telephone might be an alternative to face-to-face settings.

## Discussion

This study has provided insights into the perspectives of relevant interventions in future research. In line with previous studies [11–13, 31], the results illustrate the need to test an information intervention providing supplemental information to pregnant couples following a diagnosis of CHD. A previous pilot study of an offline educational CD-ROM for individuals faced with a prenatal diagnosis of CHD observed that the participants reported favorable feedback and found the intervention informative [31]. Supplemental information via the Internet has potential to be highly accessible and interactive, and is particularly current considering the increasing number of patients who use the Internet to search for health-related issues [32, 33]. The fact that many existing websites about CHD are of poor quality [34] strengthens the arguments for websites that offer trustworthy supplemental information following the diagnosis. Moreover, representatives described a need for interventions that specifically address information regarding medically induced second-trimester pregnancy terminations. This raises questions regarding continuity of care and information delivery within the Swedish health care system for women and partners who decide to terminate the pregnancy after a diagnosis of a fetal anomaly. Steps need to be taken towards development of interventions with the purpose to improve information delivery to this population.

Representatives suggested interventions that offer peer support. A number of studies have concluded that peer support is desired [4, 12, 17], appreciated [17, 35], and critical for coping [18] among individuals faced with a prenatal diagnosis of fetal anomaly. Moreover, studies indicate that expectant parents faced with a prenatal diagnosis want information about support groups and support from other parents, and are more satisfied with their experience in connection to the diagnosis when offered this information [15]. According to the findings of this study, the peer support could either be offered face-to-face or through virtual settings. Other researchers have reported needs of peer support after a pregnancy termination due to a fetal anomaly [18, 35], strengthening our findings. A number of public virtual communities exist today where persons can communicate when faced with fetal diagnoses. The persons who write messages in these communities often express appreciation of the peer support, but are occasionally faced with criticism of their decision to terminate the pregnancy [17], as is confirmed by the findings of this study. Consequently, it is possible that more confidential and secure platforms would be more appropriate alternatives. However, no robust evidence exists of virtual peer support currently, partly because previous studies have not evaluated it as a singular intervention [36]. We encourage researchers to conduct studies that investigate peer support following a prenatal diagnosis, and acknowledge that more studies are needed that focus specifically on peer support as singular interventions following a prenatal diagnosis.

This study included ten patient representatives with experience of a prenatal diagnosis, both females and males who continued or terminated the pregnancy. To the best of our knowledge, refining research topics towards more patient-focused research is a new approach in fetal cardiology. An important aspect to take into account when interpreting the results is that this study used qualitative methods to generate hypotheses about future research, not draw any generalizable conclusions. We acknowledge that the views of these representatives are individual and may be specific to the context of Sweden. Purposeful sampling was used to recruit patient representatives with maximum variation, which previous literature suggests as an appropriate strategy when PPI is used to improve relevance of research [22]. Representatives of both sexes and with varied age and number of born children were recruited. However, we failed to recruit patient representatives with varied educational backgrounds. Furthermore, the majority of the representatives were born in Sweden, and we did not collect any additional information about their cultural backgrounds. All of the patient representatives in the termination group were born in Sweden, perhaps reflected by the fact that the decision to terminate the pregnancy is associated with ethnic origin [37, 38]. We acknowledge the importance of culturally diverse groups when conducting PPI research, and the possibility that the results might not be transferable to individuals with lower educational levels and immigrants who terminate the pregnancy. We do not make any claims concerning the preferences and prioritized research topics among persons with other backgrounds and in settings that differ from the Swedish context. We also acknowledge that no parents to children with chromosomal anomalies were included as representatives. This fact may reflect that a higher proportion of pregnancies are terminated when multiple congenital anomalies are detected, in comparison to isolated anomalies [39, 40]. Finally, the findings are dependent on the context of the study, for example the availability of legal pregnancy termination. Thus, we regard the findings to be transferable to settings similar to that of Sweden. We did not explore the reasons behind why the representatives decided to participate in the consultation sessions. However, many described appreciation of the opportunity to meet and discuss with peers who shared similar experiences. On the other hand, we acknowledge the possibility that the participation of the representatives could have included a risk of psychological consequences. However, we were prepared to refer to psychosocial support within the Swedish health care system if these situations occurred, and did not observe any evidence of such personal reactions during the group discussions.

## Conclusions

Several interventions were suggested by patient representatives, indicating a need for multiple intervention studies to be conducted in the context of a prenatal diagnosis of a congenital heart defect in the fetus. In summary, we recommend the following interventions to be evaluated in future studies:

1. Supplemental information following the diagnosis and before pregnancy termination.
2. Follow-up consultations to offer repeated information.
3. Peer support during the remainder of the pregnancy or after pregnancy termination.
4. Additional professional psychosocial support.

## Abbreviations

CHD: Congenital heart defects
PPI: Patient and public involvement

## References

[1] Bergman G, Borgström E, Lundell B, Sonesson S-E (2008). Improved prenatal diagnosis of congenital heart defects. A follow-up study of prenatal ultrasound screening. Läkartidningen.

[2] Garcia J, Bricker L, Henderson J, Martin M-A, Mugford M, Nielson J (2002). Women’s views of pregnancy ultrasound: a systematic review. Birth.

[3] Lalor JG, Devane D, Begley CM (2007). Unexpected diagnosis of fetal abnormality: women’s encounters with caregivers. Birth Berkeley Calif.

[4] McCoyd JLM (2009). What do women want? Experiences and reflections of women after prenatal diagnosis and termination for anomaly. Health Care Women Int.

[5] Lalor J, Begley CM, Galavan E (2009). Recasting Hope: A process of adaptation following fetal anomaly diagnosis. Soc Sci Med.

[6] Sandelowski M, Barroso J (2005). The travesty of choosing after positive prenatal diagnosis. J Obstet Gynecol Neonatal Nurs.

[7] Wool C (2011). Systematic review of the literature: parental outcomes after diagnosis of fetal anomaly. Adv Neonatal Care.

[8] Kaasen A, Helbig A, Malt UF, Naes T, Skari H, Haugen G (2010). Acute maternal social dysfunction, health perception and psychological distress after ultrasonographic detection of a fetal structural anomaly. BJOG.

[9] Rona RJ, Smeeton NC, Beech R, Barnett A, Sharland G (1998). Anxiety and depression in mothers related to severe malformation of the heart of the child and foetus. Acta Paediatr.

[10] Howe D (2014). Ethics of prenatal ultrasound. Best Pract Res Clin Obstet Gynaecol.

[11] Carlsson T, Bergman G, Wadensten B, Mattsson E (2016). Experiences of informational needs and received information following a prenatal diagnosis of congenital heart defect. Prenat Diagn.

[12] Bratt E-L, Järvholm S, Ekman-Joelsson B-M, Mattson L-Å, Mellander M (2015). Parent’s experiences of counselling and their need for support following a prenatal diagnosis of congenital heart disease - a qualitative study in a Swedish context. BMC Pregnancy Childbirth.

[13] Carlsson T, Bergman G, Melander Marttala U, Wadensten B, Mattsson E (2015). Information following a diagnosis of congenital heart defect: experiences among parents to prenatally diagnosed children. PloS One.

[14] Menahem S, Grimwade J (2004). Counselling strategies in the prenatal diagnosis of major heart abnormality. Heart Lung Circ.

[15] Johnson J, Adams-Spink G, Arndt T, Wijeratne D, Heyhoe J, Taylor P. Providing family-centred care for rare diseases in maternity services: parent satisfaction and preferences when dysmelia is identified. Women Birth. 2016. 10.1016/j.wombi.2016.04.007 27156021

[16] Hilton-Kamm D, Sklansky M, Chang R-K (2014). How not to tell parents about their child’s new diagnosis of congenital heart disease: an internet survey of 841 parents. Pediatr Cardiol.

[17] Carlsson T, Landqvist M, Mattsson E (2016). Communication of support and critique in Swedish virtual community threads about prenatal diagnoses of fetal anomalies. BMC Pregnancy Childbirth.

[18] Lafarge C, Mitchell K, Fox P (2013). Women’s experiences of coping with pregnancy termination for fetal abnormality. Qual Health Res.

[19] Lloyd K, White J (2011). Democratizing clinical research. Nature.

[20] Tallon D, Chard J, Dieppe P (2000). Relation between agendas of the research community and the research consumer. Lancet.

[21] Petit-Zeman S, Firkins L, Scadding JW (2010). The James Lind Alliance: tackling research mismatches. Lancet.

[22] Entwistle VA, Renfrew MJ, Yearley S, Forrester J, Lamont T (1998). Lay perspectives: advantages for health research. BMJ.

[23] INVOLVE (2012). Briefing notes for researchers: Public involvement in NHS, public health and social care research.

[24] Oliver SR, Rees RW, Clarke-Jones L, Milne R, Oakley AR, Gabbay J (2008). A multidimensional conceptual framework for analysing public involvement in health services research. Health Expect.

[25] Brett J, Staniszewska S, Mockford C, Herron-Marx S, Hughes J, Tysall C (2014). A systematic review of the impact of patient and public involvement on service users, researchers and communities. Patient.

[26] Domecq JP, Prutsky G, Elraiyah T, Wang Z, Nabhan M, Shippee N (2014). Patient engagement in research: a systematic review. BMC Health Serv Res.

[27] Cheyne H, McCourt C, Semple K (2013). Mother knows best: developing a consumer led, evidence informed, research agenda for maternity care. Midwifery.

[28] Crang-Svalenius E, Dykes AK, Jörgensen C (1998). Factors influencing informed choice of prenatal diagnosis: women’s feelings and attitudes. Fetal Diagn Ther.

[29] Patton MQ (2002). Qualitative research & evaluation methods.

[30] Graneheim UH, Lundman B (2004). Qualitative content analysis in nursing research: concepts, procedures and measures to achieve trustworthiness. Nurse Educ Today.

[31] Caldera K, Ha D, Menahem S (2013). The development of a CD-ROM: an aid to fetal cardiac diagnosis and counseling. Fetal Diagn Ther.

[32] Fox S, Duggan M (2013). Health Online 2013.

[33] Kummervold PE, Chronaki CE, Lausen B, Prokosch H-U, Rasmussen J, Santana S (2008). eHealth trends in Europe 2005-2007: a population-based survey. J Med Internet Res.

[34] Carlsson T, Bergman G, Karlsson A-M, Mattsson E (2015). Content and quality of information websites about congenital heart defects following a prenatal diagnosis. Interact J Med Res.

[35] Gordon L, Thornton A, Lewis S, Wake S, Sahhar M (2007). An evaluation of a shared experience group for women and their support persons following prenatal diagnosis and termination for a fetal abnormality. Prenat Diagn.

[36] Eysenbach G, Powell J, Englesakis M, Rizo C, Stern A (2004). Health related virtual communities and electronic support groups: systematic review of the effects of online peer to peer interactions. BMJ.

[37] Tararbit K, Bui TTT, Lelong N, Thieulin A-C, Goffinet F, Khoshnood B (2013). Clinical and socioeconomic predictors of pregnancy termination for fetuses with congenital heart defects: a population-based evaluation. Prenat Diagn.

[38] Chenni N, Lacroze V, Pouet C, Fraisse A, Kreitmann B, Gamerre M (2012). Fetal heart disease and interruption of pregnancy: factors influencing the parental decision-making process. Prenat Diagn.

[39] Calzolari E, Barisic I, Loane M, Morris J, Wellesley D, Dolk H (2014). Epidemiology of multiple congenital anomalies in Europe: a EUROCAT population-based registry study. Birth Defects Res A Clin Mol Teratol..

[40] Dolk H, Loane M, Garne E, European Surveillance of Congenital Anomalies (EUROCAT) Working Group (2011). Congenital heart defects in Europe: prevalence and perinatal mortality, 2000 to 2005. Circulation.

